# Vision and Deep Learning-Based Algorithms to Detect and Quantify Cracks on Concrete Surfaces from UAV Videos

**DOI:** 10.3390/s20216299

**Published:** 2020-11-05

**Authors:** Sutanu Bhowmick, Satish Nagarajaiah, Ashok Veeraraghavan

**Affiliations:** 1 Department of Civil and Environmental Engineering, Rice University, 6100 Main Street, Houston, TX 77005, USA; sb70@rice.edu; 2 Department of Mechanical Engineering, Rice University, 6100 Main Street, Houston, TX 77005, USA; 3 Department of Electrical and Computer Engineering, Rice University, 6100 Main Street, Houston, TX 77005, USA; vashok@rice.edu

**Keywords:** computer vision, morphological operations, unmanned aerial vehicle, U-Net

## Abstract

Immediate assessment of structural integrity of important civil infrastructures, like bridges, hospitals, or dams, is of utmost importance after natural disasters. Currently, inspection is performed manually by engineers who look for local damages and their extent on significant locations of the structure to understand its implication on its global stability. However, the whole process is time-consuming and prone to human errors. Due to their size and extent, some regions of civil structures are hard to gain access for manual inspection. In such situations, a vision-based system of Unmanned Aerial Vehicles (UAVs) programmed with Artificial Intelligence algorithms may be an effective alternative to carry out a health assessment of civil infrastructures in a timely manner. This paper proposes a framework of achieving the above-mentioned goal using computer vision and deep learning algorithms for detection of cracks on the concrete surface from its image by carrying out image segmentation of pixels, i.e., classification of pixels in an image of the concrete surface and whether it belongs to cracks or not. The image segmentation or dense pixel level classification is carried out using a deep neural network architecture named U-Net. Further, morphological operations on the segmented images result in dense measurements of crack geometry, like length, width, area, and crack orientation for individual cracks present in the image. The efficacy and robustness of the proposed method as a viable real-life application was validated by carrying out a laboratory experiment of a four-point bending test on an 8-foot-long concrete beam of which the video is recorded using a camera mounted on a UAV-based, as well as a still ground-based, video camera. Detection, quantification, and localization of damage on a civil infrastructure using the proposed framework can directly be used in the prognosis of the structure’s ability to withstand service loads.

## 1. Introduction

Condition assessment of civil engineering structures for its safety and remaining lifetime has been in focus for the past couple of decades. Mostly, it consisted of harnessing dynamic response by attaching acceleration and displacement sensors with further post-processing of the data to evaluate the presence of damage in those structures. This method provides the global behavioral pattern of the structure which may sometimes provide local damage indications depending on the kind of structure and the spatial density of the sensors.

On the other hand, local assessment of structural damage is mostly carried out by visual inspection by experts. Otherwise, by placing contact-based strain sensors or by carrying out acoustic-based non-destructive testing. Sun et al. [1] present a detailed review of structural health monitoring methods based on the big data and artificial intelligence algorithms for bridges, as well as identifies challenges associated with them. However, given the size of the structure itself, the whole process becomes time-consuming and prone to human errors. In addition, in some regions of the structure, it is difficult for human beings to gain access. Contact-based sensors also suffer from high maintenance due to the wrath of the exterior environment they are exposed to.

Recent advances in the application of non-contact camera-based structural health monitoring got huge incentive with the development in high-resolution cameras coupled with robust computer vision algorithms. Some of the real-life applications of such computer vision algorithms include face detection in mobile cameras, motion detection for surveillance, traffic sign, and pedestrian detection in autonomous cars. Similar technologies can be customized to detect local damages on structural surfaces using video measurements. Video acquired from surveillance cameras installed on important structures, like bridges, is a valuable source of data for damage detection. Moreover, fast and automatic damage inspection of large scale structures can be performed using the unmanned aerial vehicle (UAV) equipped with a digital camera and onboard microprocessor. Koch et al. [2] present a review of computer vision algorithms that have been used for damage detection and condition assessment of civil infrastructure.

The previously reported methods for detecting cracks on concrete surfaces from its image can be broadly classified into two categories: image processing and machine learning. Some of the earlier works involve application of image processing techniques to identify the edges in the image which corresponds to presence of crack patterns. Among various methods of edge detection from images, like Sobel filter, Canny edge detector, Fast Fourier Transform (FFT), and Fast Haar Transform (FHT), Abdel-Qader et al. [3] found FHT to be superior among them. Yamaguchi and Hashimoto [4] identified crack pixels in an image using the technique of iterative percolation. Hutchinson and Chen [5] obtained the optimal thresholding parameters of Canny edge detection and FHT using Bayesian decision theory.

Methods based on only image processing rely on the efficient extraction of predefined visual crack features, which does not require supervised learning using extensive labeled image data. Image processing involves extracting preset features from an image that can visually identify damages in structures in a qualitative sense. However, the methods are less robust as the preset features that are looked for do not encompass extensive variability that real images offer, like luminescence, surface texture, shadow, scale, and rotation invariance, as well as noise content. A large amount of dataset in form of images of both cracked and uncracked concrete surfaces is utilized to extract quantitative features. Such a bag of features, along with the binary labels of cracked or uncracked, are used to train a classification model that can predict whether an image of the concrete surface has the presence of cracks or not. Jahanshahi et al. [6] trained nearest-neighbor model, a polynomial kernel support vector machines (SVMs) model, and an artificial neural network model for binary image classification using morphological features of the detected objects. Chen et al. [7] trained an SVMs binary classification model using local binary patterns (LBP) as features. Concrete surfaces consist of minor cracks due to its thermal expansion. Several kinds of superficial breathing cracks are also present on its surface which opens and closes due to varying loads. The continuous monitoring of structures subjected to cyclic loadings [8] will provide valuable insights not only regarding deterioration and fracture processes but also in the planning of maintenance and repair. Therefore, the mere detection of cracks is not sufficient for damage detection. It is imperative to constantly monitor concrete surfaces for crack initiation and its propagation. Yang and Nagarajaiah [9] proposed the method of dynamic tracking of damages on concrete surfaces. Bhowmick and Nagarajaiah [10] further developed the method to detect and quantify multiple damages on concrete surface from its video.

The previously described methods of binary classification using machine learning depend on the extraction of relevant features from the images. This step of feature engineering is skipped while using methods based on deep convolution neural network (D-CNN) as it can adaptively learn relevant features of the images in its training phase.But, the main benefit of D-CNN models is the significant improvement of the accuracy they provide over the other machine learning methods [11,12,13], given large labeled image dataset is available. Cha et al. [14] trained a D-CNN model using 40 k manually annotated images of dimension 256×256×3 for detecting the presence or absence of cracks in images of concrete surfaces. Chen and Jahanshahi [15] used 5326 manually annotated images of pixel size 120×120×3 which were extensively augmented to increase the data size to more than 250 k by rotating, flipping and varying brightness of the images to train D-CNN which was augmented with Naive Bayes classifier to separate out cracks from non-cracks using results of consecutive frames of a video. In both the methods, the trained network takes as input an image of the concrete surface of the specific pixel size as it is trained for and outputs a label of crack or non-crack. For an image of a concrete surface larger than the image size with which the network is trained, the method first extracts cropped image patches of required dimension using the technique of sliding window. The individual extracted patches are further predicted as damaged or not; if damaged, then a square bounding box is drawn around the patch in the original image. Thus, for a large image of a concrete surface or video sequence, the detected cracks are marked with a bounding box. The results on the validation set show excellent performance of the classification algorithm, but the method fails to reveal more information such as length, width, and area of the crack apart from its detection.

Although the technique of image classification using CNN could satisfactorily detect the presence of cracks by generating a bounding box, further information required to quantify the damage could not be processed from the obtained bounding box. Hence, the technique of image segmentation is utilized to classify the presence of cracks in the pixel level of an image. Very recently, deep neural network architectures are trained using pixel-level annotated images of concrete cracks to obtained an image segmentation model of concrete cracks. Ni et al. [16] trained a modified GoogLeNet architecture [17] with hand-labeled images of concrete cracks to propose a crack segmentation model. Kim and Cho [18] trained a mask region-based CNN with hand-labeled concrete crack images to obtain a crack segmentation model and further measured the crack width of cracks present in a concrete wall using the output of the model. Liu et al. [19] proposed DeepCrack, a deep neural network architecture for segmentation of concrete cracks. Liu et al. [20] used U-Net architecture [21] for image segmentation and showed that U-Net can achieve higher accuracy score of crack segmentation with less number of training images compared to previously reported segmentation networks. The pixel-level labeling of cracks from the concrete surface image is time-consuming and labor-intensive work. Hence, it is beneficial to use a CNN algorithm that requires less training set images to achieve high accuracy in segmentation score. In this study, the U-Net architecture is chosen for obtaining an image segmentation model that needs less number of pixel-annotated ground truth images to achieve high segmentation. The model is trained using only 346 images obtained from two different sources which include both the training and validation set, but the dataset is expanded using data augmentation to add variability in the training images. The efficacy and robustness of the trained model are experimentally verified on an 8-foot-long concrete beam pseudo-statically loaded till its failure. The whole experiment is video recorded using a camera mounted on a UAV. The video is processed using the trained segmentation model which detects the initiation and propagation of multiple cracks at different instants of time on the concrete beam. The image of the concrete beam used in experimental verification does not form part of the dataset used to train and validate the U-Net segmentation model. This makes the trained U-Net model completely unbiased towards the outcome of the segmentation results, as well as confirms the robustness of the model in segmenting unseen images of concrete surfaces in different environmental conditions. The segmented images of cracks are further processed to obtain geometric measurements of the cracks as they evolve with time.

The output of an image segmentation model to detect crack is also an image. Hence, further processing of the predicted image is needed to obtain the geometrical quantities which are essential for addressing condition assessment of concrete structures. Zhu et al. [22] proposed morphological operations on crack map of concrete surface to obtain geometrical properties of cracks. Jahanshahi et al. [6] proposed similar morphological operations to compute crack properties on concrete surface. Adhikari et al. [23] proposed a method of retrieving crack length and width from its image. The geometrical properties of cracks are converted to physical units using a scaling factor obtained from a known dimension of a structural element within the acquired image [6,18]. However, the proposed operations are designed to compute length and the maximum width of single crack from an image of crack present in the concrete surface. In this paper, the crack quantification methodology extends to detect multiple cracks in each frame of the UAV video of the concrete surface. The length of individual cracks is measured, as well as the dense measurement of its width, along the length of the crack. The maximum crack width and its location in the image are identified in the image from the spatially dense measurement of crack width, along its length. The previous studies have fitted a straight line to the skeleton of the crack to measure its orientation. But, in real-life scenarios for concrete structures, one crack may divide to form multiple others, or several small cracks may join together to form a single crack. During the inspection of concrete structures, an expert infers the nature of crack from its dominant orientation, i.e., whether it is flexure or shear crack. Hence, the simple fitting of a straight line to those complex cracks does not provide a dominant orientation of the cracks, in turn, it does not provide required inference about the nature of the crack. In this study, the dominant orientation of multiple cracks are extracted individually and quantified such that proper inference about the cracks nature is possible. The method could track the evolution and propagation of multiple cracks by quantifying individual crack’s length, width, area, and dominant orientation. The qualitative nature of cracks is inferred using the measured dominant orientation of cracks throughout the course of the experiment, which confirms the evolution of initial flexure cracks to the formation of shear cracks near failure. To the best knowledge of the authors, such dense quantitative and qualitative inference of multiple crack propagation and evolution on a concrete surface from UAV video has not been presented before.

The final aim of implementing damage detection algorithms where input is an image of the concrete surface is to automate the process of structural inspection using Unmanned Aerial Vehicle (UAV) equipped with a video camera. But, some practical considerations need to be addressed in the overall framework from video acquisition to the quantification of damage. The video frames will not only capture the concrete surface but also contain other objects and background. In addition, mere detection of damages will not be enough if its quantification is required to complete the condition assessment of the structure. We propose the following steps for automatic detection of cracks and quantification of damage of concrete structure as an efficient substitute to manual inspection:

1. Monitoring of concrete structure using video measurements from high resolution camera mounted on UAV equipped with LiDAR (Light Detection and Ranging) system which can be used to create 3D mapping of the whole structure.
2. Segmentation of pixels belonging to structural surface from non-structural objects in each frame of the video.
3. Within pixels belonging to concrete surface, further segmentation of damages, like crack or spalling of concrete from non-damaged concrete surface pixels.
4. Quantification of the geometric properties of damages, along with its localization from the 3D mapping of the structure, will provide sufficient information to assess condition of the structure.

**Figure 1:**
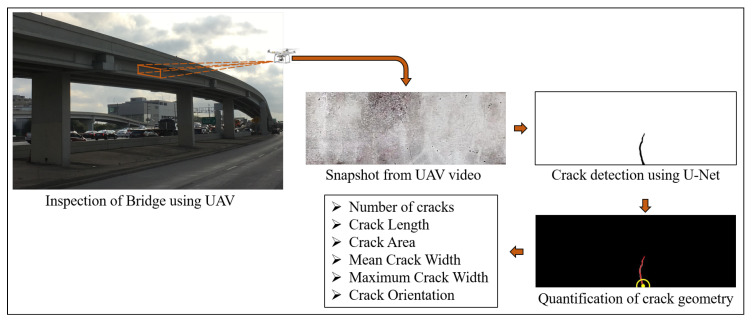
Outline of the proposed framework: Each frame of video acquired using the Unmanned Aerial Vehicle (UAV) is processed using U-Net to detect presence of crack at pixel level, which is further processed to obtain the geometric properties of the detected crack.

The suggested steps detect the presence of damage, localizes it with respect to the overall structure, further quantifies the damage based on its dimensions and location. This paper address steps 3 and 4 of the suggested framework instead of proposing a solution for the whole presented framework. The paper proposes a methodology to automatically detect and geometrically quantify cracks on the concrete surface from its video captured using UAV. The first two steps of the framework are beyond the scope of this study and are the subject of a future study. Hence, the concrete surface is tracked over subsequent frames of the video to automatically select the region of interest (ROI). The ROI is selected from the first frame of the video and the region is tracked over multiple frames of the UAV video even when the UAV is translated. The outline of the proposed framework is illustrated in Figure 1 which uses video recorded using UAV for automatic bridge inspection to detect cracks, as well as quantify its geometric features. The paper proposes a Convolution Neural Network (CNN)-based segmentation algorithm to classify pixels belonging to cracks from images of concrete surface which only uses 205 images, along with its pixel-level annotated ground truth binary image, for its training and validation. The algorithm takes as input the image of the concrete surface and yields a binary image as output with pixels belonging to cracks, if present, having values of ones, and pixels containing uncracked concrete surface having zero values. The obtained binary image of the crack is required to obtain the geometric characteristics of the cracks, like length, area, width, and orientation of individual cracks, using image processing techniques. The obtained geometrical features are in pixel units which are converted to physical units using a scaling factor obtained using the known physical dimension of the structural element. The method extends beyond mere detection of cracks as it quantifies the amount of damage depending on its geometric properties. The proposed method is successfully able to detect and quantify cracks on the beam used for experimental validation from its recorded video.

The key contribution of the paper consists of experimentally validating the proposed method of detecting and quantifying cracks on the concrete surface from video obtained using UAV. The proposed methodology consists of training U-Net deep network architecture with a small number of the dataset obtained from two different sources. Then, the segmented image of the crack is processed to obtain spatially dense geometric measurements of cracks consisting of length, width, area and dominant orientation of individual cracks. The initiation, propagation, and evolution of individual cracks of the pseudo-statically loaded 8-foot-long beam for experimental validation are quantified at the pixel level, as well as in time. The obtained information is further used to infer the nature of crack formation. The method validates the formation of initial flexure cracks and then its evolution to shear cracks. To the best knowledge of the authors, this is the first instance of presenting the efficacy of crack detection, quantification, and inference of its propagation and evolution, using UAV videos. First, an image segmentation deep neural network is trained with a very small number of concrete surface images (346 pixel-annotated images). The binary crack image is processed to compute its geometrical properties, like length, width, area, and dominant orientation of the cracks, which helps in recognizing the nature of the crack, i.e., whether the crack is flexure or shear crack. Further, experimental validation of the proposed method includes the application of the trained image segmentation algorithm to detect the presence of cracks on a concrete beam using video recordings from UAV, as well as using a ground-based camera. The images used for training the segmentation network does not contain the images of the beam experiment. Hence, the result of the experiment validates the robustness of the trained model in detecting cracks at a pixel scale of different images of the concrete surface obtained using different sources and under a different condition that is not part of training data. Section 2 describes the deep learning algorithm used for the segmentation of cracks from images of concrete surface. The deep neural network is trained using images of the concrete surface, along with its pixel-level annotated ground truth, the details of which are provided in Section 3. Hyperparameters of the network are tuned using images from the validation set, which are kept separate from the training set as discussed in Section 4. The quantification of the geometric properties of a crack is proposed in Section 5 using morphological operations on binary images of crack objects. The details of the experimental validation of the suggested method are provided in Section 6.

## 2. Deep Segmentation Network

**Figure 2:**
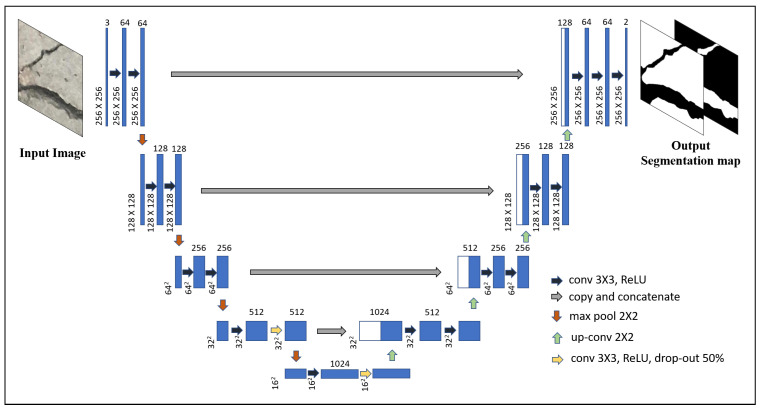
Deep Convolution Neural Network architecture, U-Net, with sample RGB input image and output segmentation maps which denotes probability of crack and non-crack pixels.

The Deep Learning architecture used in this paper, U-Net [21], has proved effective in segmenting biomedical images where the amount of pixel annotated images are limited. It is a difficult and time-consuming process to gather images of concrete cracks, as well as manually construct ground truth of each image. But, the number of images used during training is further expanded by augmenting the dataset with random rotations and flipping both horizontally and vertically each original image as suggested in Reference [21]. The details of the network architecture are shown in Figure 2. The unique part of this CNN architecture consists of a contracting path followed by an expansive path. The adopted architecture is almost similar to the one suggested by Ronneberger et al. [21] with small modifications. The input to the original U-net architecture consists of a grayscale image, or D=1 for input image shape of [W×H×D] where *W*, *H*, and *D* are the width, height, and depth of the image. But, in the modified architecture, the RGB image is used as input where D=3. In addition, W=D=256 for input image in the modified architecture instead of W=D=572 in the original one. The contracting part starts off with two 3×3 zero padded convolutions such that the output spatial dimension remains the same as the input but a number of feature map is extended to 64. Each convolution is followed by a rectified linear unit (ReLU) non-linear activation function. The feature maps are downsampled by carrying out 2×2 max pooling operation with stride 2. The steps are further repeated and each time after downsampling spatial dimension by max pooling, the number of feature maps is doubled in the subsequent convolution step. At the last two steps of the contraction side, drop out layer is added. In the expansive path, at each step, the spatial dimension is doubled by upsampling followed by 2×2 convolution (up-convolution), then the feature maps are doubled by concatenating corresponding feature maps from the contracting path, further carrying out 3×3 convolutions two times with ReLU activation. The final layer consists of 1×1 convolution layer with sigmoid activation to reduce the number of feature maps from 64 to a number of classes for pixel level classification. The zero padded convolutions ensure the output segmentation map is of the same size as that of the input image.

Both the input images and their pixel-level annotated ground truth are needed to optimize binary cross-entropy loss function which is computed by pixel-level softmax over the final feature map computed as.

(1) $$p_{k}\left(x\right)=\frac{exp(a_{k}\left(x\right))}{\sum_{k^{\prime }=1}^{K}exp\left(a_{k}^{\prime }\left(x\right)\right)},$$

where $a_{k}\left(x\right)$ is the activation in the *k*th feature map at the pixel position *x* and *K* is the total number of classes. The cross entropy loss function is computed using the ground truth of the image as

(2) $$L_{ce}=\sum_{x\in \Omega }o_{k}\left(x\right)log\left(p_{k}\left(x\right)\right),$$

where Ω={1,2,…,K}, and $o_{k}\left(x\right)$ is the ground truth of *k*th class at pixel *x*. $L_{ce}$ is minimized using Adam optimization with adaptive learning rate. The weights of the convolution layers are initialized using He normal [24].

## 3. Dataset

**Figure 3:**
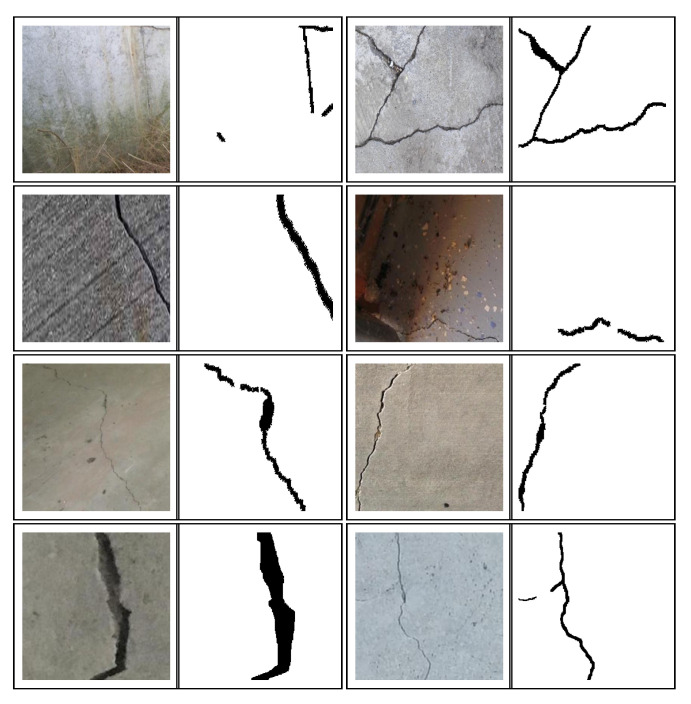
Sample RGB images from training set and their pixel-level annotated binary ground truth images denoting crack pixels.

Images of the concrete surface with cracks are obtained from a partial crack dataset [25]. The dataset consists of 136 RGB images of cracks in the concrete surface having a pixel resolution of 768×768. It also contains hand-labeled pixel-level annotated ground truth of the cracks which are binary images having zeros in the pixels belonging to the crack and ones in the rest of the concrete surface. The images are cropped to 4 different images re-scaled to 256×256 pixels, as well as flipped horizontally and rotated by 180 degrees. Sixty-nine RGB images of cracked concrete surface are randomly selected from concrete crack images for classification [26] each of size 385×385 pixels. Those pixel-level ground truth images of cracks are hand-labeled using the Image Labeler app in MATLAB 2018a [27]. The RGB images, along with the corresponding ground truths, are re-scaled to 256×256 pixels, along with augmenting the dataset by rotating the images at an angle of 90, 180, and 270 degrees, along with flipping it horizontally and vertically. The final size of the dataset consists of 2046 augmented images, out of which 1700 are randomly selected for training the segmentation network, and the remaining 346 images form a validation set which is used to search for optimal hyper-parameters. Further, during the training of the network in batches, the images are randomly rotated by a maximum of 20 degrees, shifted horizontally, vertically and zoomed by 5 percent of actual dimension, as well as undergone sheer transformation by 0.05 radians. This random transformations during training in batches of a small number of images not only augment the training data size but also brings in the real-life variability, which helps in making the model robust. Some of the images from the training set are shown in Figure 3, along with its corresponding binary pixel-level ground truth. In total, only 205 RGB images with its corresponding pixel-level annotated ground truth images are used to train and validate the network with the help of extensive data augmentation. The U-Net deep neural network architecture is implemented in Tensorflow [28] using one of its API, Keras [29]. The model is trained using the NVIDIA GTX 1070 Ti Graphical Processing Unit (GPU) with 8 GB memory.

## 4. Prediction of U-Net

The two hyper-parameters chosen to optimize U-Net are batch size i.e., number of images used per iteration and number of epochs i.e., the total number of times all the images are used for optimizing parameters of the deep network. The output of U-Net is not a binary image, but a probability map having values between 0 and 1. Pixels that are more probable to belong to cracks have scores close to 0 and pixels which are more probable to be un-cracked concrete surface have values close to 1. Hence, the probability map needs a threshold set in order to convert it into a binary image. Otsu’s thresholding method [30] is used to generate binary images. Initially, the threshold level is set to 1.0 to find the optimal hyper-parameters. The performance of the hyper-parameters are assessed based on Intersection-Over-Union (IoU) score of the crack pixels. *IoU score*, also known as the Jaccard similarity coefficient, for each class is the ratio of the number of correctly classified pixels to the union of ground truth pixels and predicted pixels of that class.

(3) $$IoUscore=\frac{TP}{TP+FP+FN},$$

where true positives (TP) represents the overlapped pixels of the predicted mask with the ground truth mask of the crack, and false negatives (FN) are pixels of the ground truth mask which are not predicted by the algorithm and false positives (FP) are incorrectly labeled pixels of the predicted mask. The validation set is used to compute the *IoU scores* of crack for different values of the hyper-parameters as shown in Table 1. Due to the limitation on GPU memory size and growing computational cost with the added number of epochs, batch sizes of 5 and 10 are considered with incrementally varying the corresponding number of epochs till IoU score on the test data peaks, keeping the threshold level constant. The batch size and number of epochs are selected as 10 and 30 respectively.

**Figure 4:**
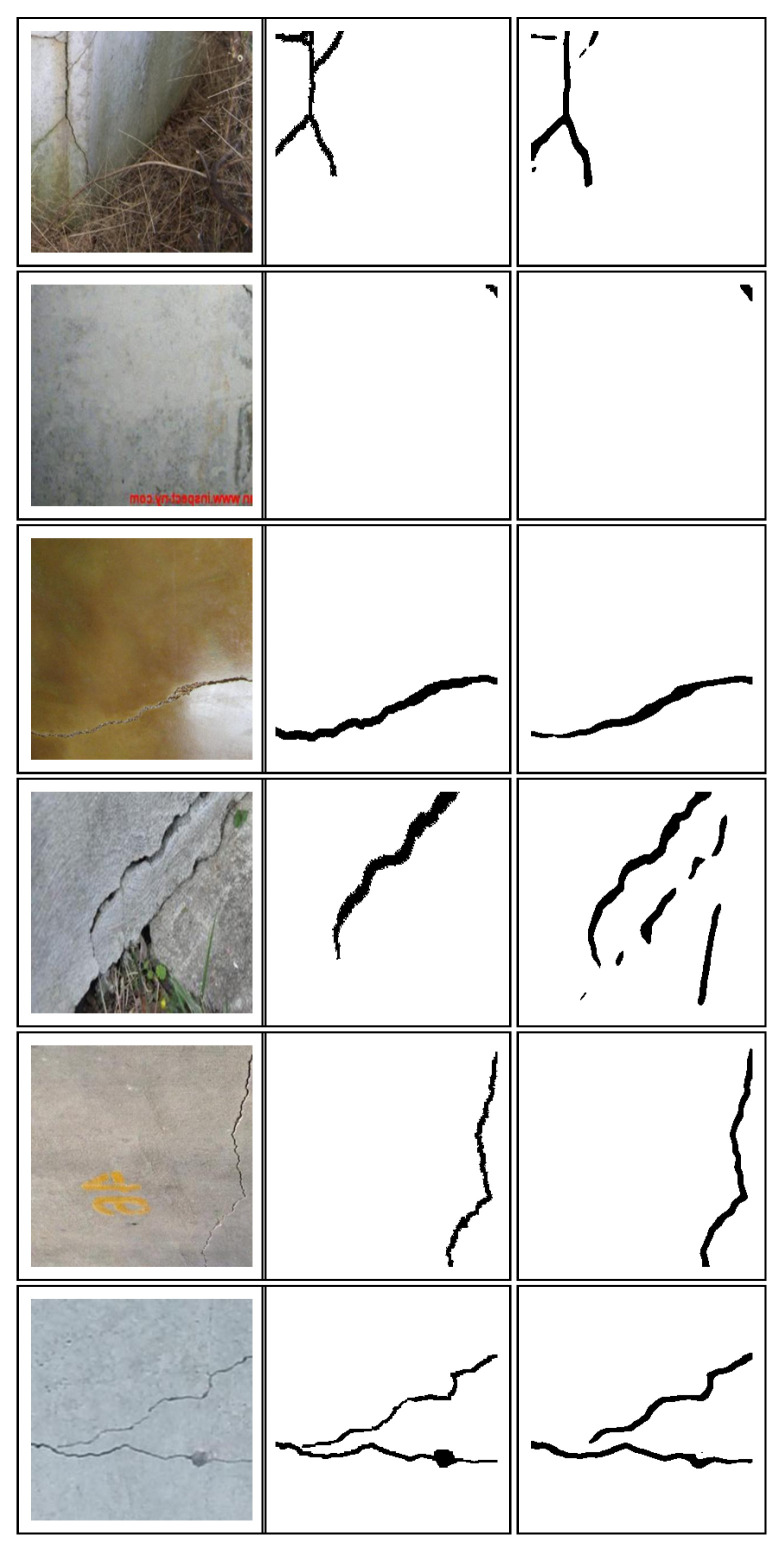
Sample RGB images from validation set (**left** column) with corresponding ground truths (**middle** column) and predicted binary image of the crack (**right** column).

Further, the threshold level in Otsu’s method is varied to find out the effect of it on the *IoU score* of a crack mask as shown in Table 2. The threshold level has a major effect on the *IoU score*. The value of 0.80 is chosen as it results in the best *IoU score*. Some of the predicted images of the algorithm are shown in Figure 4, along with its ground truth images.

## 5. Crack Characterization

The output of the algorithm so far provides a binary image to highlight crack present in the concrete surface. However, accessing the amount of damage, along with the subsequent decision of retrofitting, depends on the location of the crack, as well as its geometric properties, like its length, width, orientation, number of cracks, etc. This paper focuses on quantifying the geometrical properties of concrete surface cracks, by the use of morphological operations from image processing [6,22,23].

**Figure 5:**
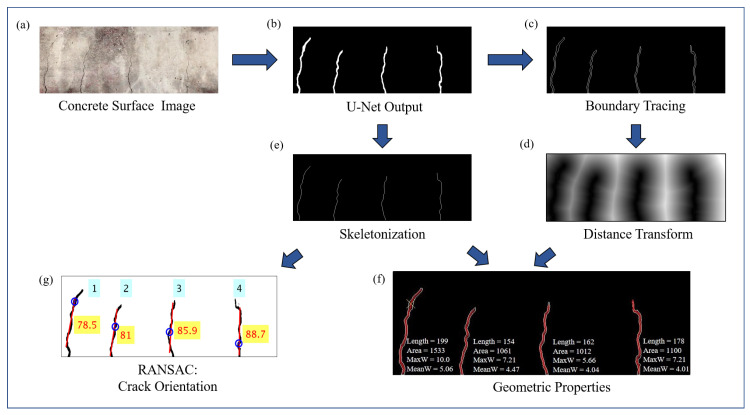
Flowchart of algorithms applied on input concrete surface image to obtain geometric properties of cracks.

Figure 5 shows the flowchart of algorithms used to obtain pixel level information about crack geometry from the concrete surface image. The image in Figure 5a is of the concrete surface with cracks, and Figure 5b shows the output of trained U-net after applying Otsu’s thresholding method. The first step in the morphological operation involves finding out the pixel area of individual cracks in the binary image. A single crack in the binary image is object formed by continuous pixels, and if there is a gap of even a single pixel between the binary objects, then its a different crack. In order to filter out small blobs which may appear in the predicted binary image due to surface textures, a threshold for pixel area of individual cracks is set below which all the small objects are removed. This image is further processed to obtain single pixel thick outline of individual binary objects (cracks) as shown in Figure 5c using boundary tracing morphological operation, as well as single pixel thick skeleton of the objects, as shown in Figure 5e, which represents the center line of the cracks. Euclidean distance transform of the outline image shown in Figure 5c is constructed as shown in Figure 5d. Distance transform of a binary image provides another image in which pixel values represent the distance in pixels to the nearest non zero pixels. Hence, any particular pixel of the image in Figure 5d represents its shortest distance to outline of cracks as represented in Figure 5c. Its values corresponding to the center-line pixels shown in Figure 5e provide the half width of cracks along the length of the individual cracks. Combining information from both Figure 5e and Figure 5d, the length and width in units of the pixel of the cracks, along its length, is obtained. The pixel area of individual cracks are obtained from Figure 5b. The geometric properties of the cracks are obtained in units of pixels which can be transformed into physical units using the depth information of the camera from the concrete surface.

In order to separately detect the qualitative orientations of individual cracks (even for more than one dominant orientation for a single crack), Random sample consensus (RANSAC) [31] algorithm is used on the skeleton pixels of individual cracks as shown in Figure 5e. It is an iterative algorithm that looks for the best fitted straight line which passes through randomly chosen two points in every iteration. Perpendicular distance of all the points from the selected line is computed and compared against threshold distance in order to classify each pixel as inliers or outliers. The steps are iterated for a certain number of sample size and the model corresponding to the best inlier ratio is selected in the end. In this paper, the RANSAC algorithm is used in succession to find dominant crack orientations which can even be more than one for certain cracks as mentioned before. After applying RANSAC for the first time on a particular crack, the slope of the best-fitted model gives the most dominant crack orientation. If the outlier ratio is more than 0.2 (selected based on heuristics), the RANSAC algorithm is applied again on the remaining outlying points successively till the overall outlier ratio is less than 0.2, each time the slope of the best fitted straight line model on the residual pixels gives the next dominant orientation. The result of the algorithm provides crack orientation as shown in Figure 5g.

## 6. Experimental Validation

### 6.1. Experimental Setup

**Figure 6:**
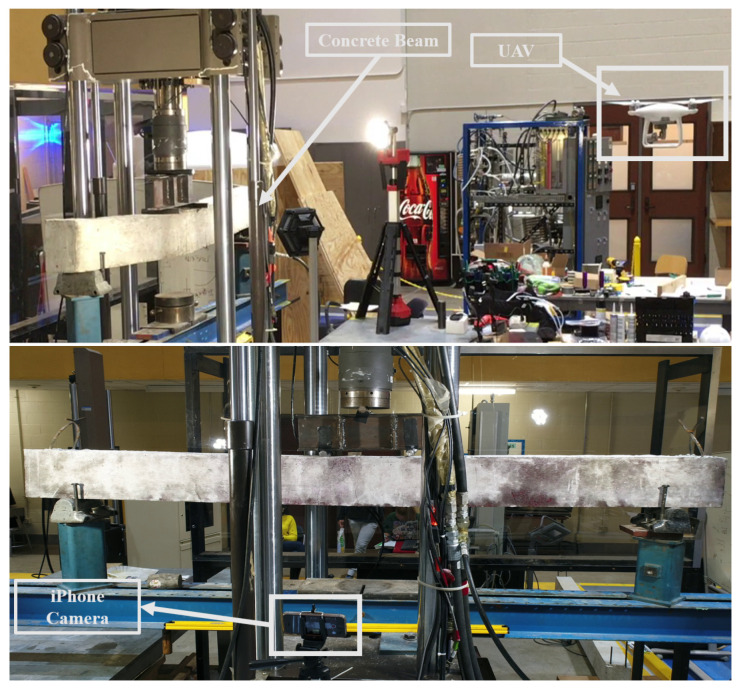
Experimental Setup showing (above) simply supported concrete beam transversely loaded at two points at its center and the video of the experiment being acquired using UAV, (below) and a still ground-based iPhone camera on a tripod is used to record the same experiment.

In the previous sections, the framework for computing geometric properties of the cracks from images of the concrete surface is proposed. In order to validate the efficacy of the proposed framework, a four-point bending test is carried out on an under-reinforced concrete beam of length 8 feet, depth of 8 inches, and a width of 6 inches. The setup of the experiment is shown in Figure 6. The video of the entire experiment is recorded using a still iPhone camera placed on a tripod and a UAV (DJI Phantom 4 Advanced) is flown close to the beam, as shown in Figure 6. The DJI Phantom 4 Advanced is calibrated for its inbuilt compass and remote controller before the flight following the steps provided in its manual [32]. During the test, the UAV is translated and rotated in all three directions in order to replicate the real world use of UAV for structural health monitoring near a structure. The video recorded using UAV has a resolution of 2160×4096 pixels at a rate of 24 frames per second (fps). The resolution of the iPhone video is 720×1280 pixels, recorded at 30 fps. The beam is gradually loaded such that it fails in flexure in the midspan. The whole experiment went on for about 30 min, and both the videos are down-sampled to 1 fps as the rate is sufficient for real-time detection of cracks.

### 6.2. Framework of Analysis

**Figure 7:**
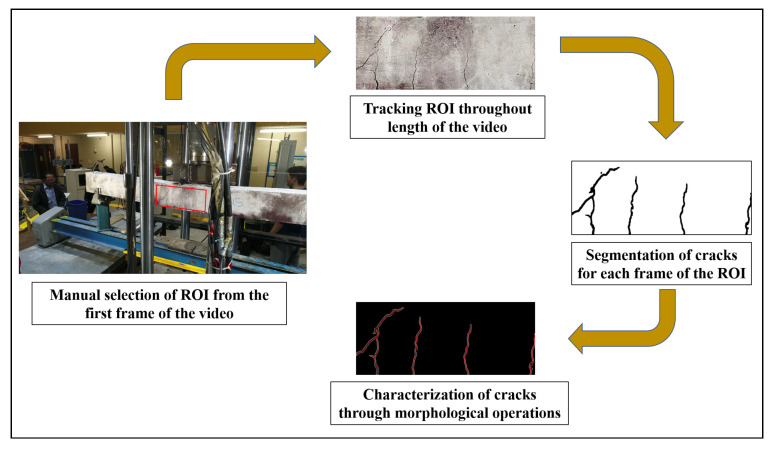
Flowchart of steps used in post-processing of recorded video frame by frame.

As mentioned in Section 1 each frame of the video needs to be first processed to segment out regions containing concrete surface; this step is a separate study for the future. For the present demonstration of the current framework, the concrete region is tracked frame by frame by first selecting the Region of Interest (ROI) as a rectangular box from the very first frame of the video, which covers the midspan of the beam where the appearance of flexure cracks are more prominent. The ROI is tracked throughout the length of the video using Kanade-Lucas-Tomasi (KLT) feature tracking algorithm [33,34,35]. Hence, irrespective of the displacement of the object (in this case the concrete surface near midspan), or translation and rotation of the camera, an image of the concrete surface per frame is extracted as defined by the ROI from the first frame of the video, over the duration of the video. The images are then processed using the same framework as discussed before also shown in Figure 7. The geometric properties of the crack, if detected, in any frame are obtained following the framework.

### 6.3. Quantification of Crack Geometry

#### 6.3.1. Crack Length, Area, and Width

**Figure 8:**
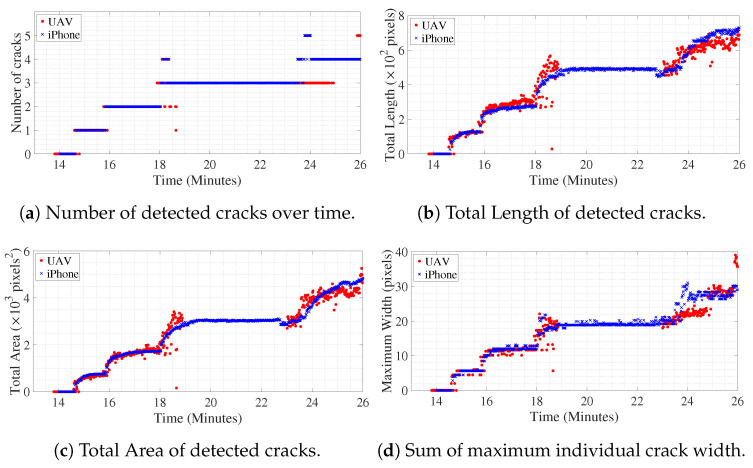
Variation of crack characteristics with time (red filled-in circles for UAV video and blue crosses for iPhone video).

**Figure 9:**
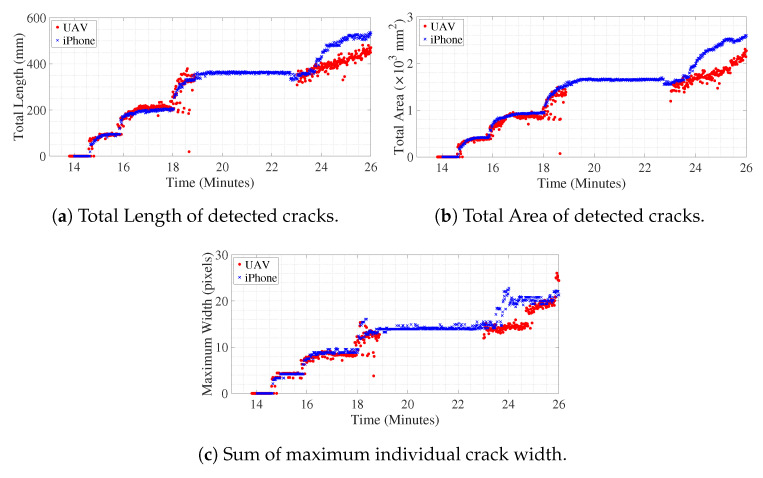
Variation of crack characteristics with time scaled to physical units (red filled-in circles for UAV video, and blue crosses for iPhone video).

Figure 8a shows the number of cracks detected in the ROI over time. The cluster of pixels classified as belonging to crack neighboring each other are identified as single crack, but, if a detected pixel is separated by more than one non-crack pixel, then it belongs to another crack. For example, in Figure 7, the number of detected cracks is four. Figure 8b,c shows the progress of total length and total area of detected cracks over time in pixel units. Along the center skeleton line of individual cracks, the width of the crack for each pixel, along the skeleton line, is computed, which gives dense geometrical information of each crack for every frame. In Figure 8d, the sum of the maximum width of individual detected cracks over time is shown. In this study, the units are approximately converted to millimeters as the depth of the beam is known in physical units. The approximation is valid till the plane of the object (in this case the concrete surface that is tracked over time) is parallel to the image plane of the camera. Hence, multiplying with the factor which converts pixels to mm, the variation of geometric measurements of the cracks is shown in Figure 9. The geometric measurements represented in physical units for the iPhone camera are reasonably accurate with some margin of error as it is kept still throughout the experiment, therefore the assumption of the plane of the object and plane of the image being parallel is valid. Initially, the UAV is kept hovering facing the surface such that the assumption can be said to be approximately correct, but, in the later part of the experiment, around 24.0 min onwards, the UAV is rotated and translated such that the plane of the object becomes skewed with respect to the plane of the image; hence, subsequent geometric measurements from UAV converted to physical units will have considerable error which is evident from deviation of the values compared to the measurements from iPhone after around 24.0 min. Tracking of objects in motion adds noise to the image, the amount of which is directly proportional to the amount of relative motion of the object between two frames. In the case of the iPhone camera which is placed on a tripod throughout the experiment, the relative motion of the concrete surface ROI is almost negligible, which only encompasses the slow vertical downward movement of the tracked object due to downward deflection of the beam. However, for video recorded from UAV, for initial 18 min of the experiment, it was kept hovering at a particular spot, afterward, the UAV and its camera were translated and rotated at times, in order to replicate the real-life health monitoring practice at the field. The noise in the measurement of geometric properties with video from UAV after 18 min is due to the relative motion of the object (in this case, the concrete surface ROI) between successive frames using the KLT feature tracking algorithm.

**Figure 10:**
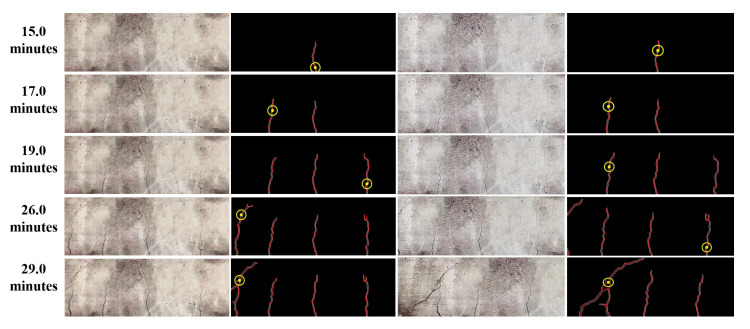
Snapshots of the original transformed images of the concrete surface and its corresponding detected cracks using the proposed framework at various instants of time with position of maximum crack width shown as yellow circle. The first and second column corresponds to video captured using iPhone, whereas the third and fourth columns corresponds to video captured using UAV.

**Figure 11:**
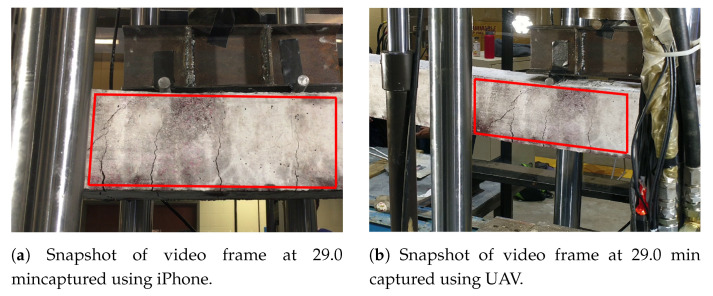
Original frame of the video at 29.0 min recorded using iPhone and UAV.

The initial crack became visible around 14.5 min after the start of the experiment. The experiment is paused between 19.4 min to 23.5 min in order to change the battery of UAV. However, the iPhone video was still recording, which is apparent from the plot of Figure 8 or Figure 9. Both in Figure 8 and Figure 9, discrete jumps in the geometric measurements of detected cracks correspond to the appearance of new cracks at those instants of time as shown in Figure 8a. Figure 10 shows frames of the video at time instants mentioned at corresponding left side of the images at each row both for iPhone video (1st column of images) and UAV video (3rd column of images) with corresponding image which shows detected cracks in red and yellow circle and dot at the position of maximum width of the crack. The images at each frame of both the videos obtained during the experiment are not part of the training or validation set of U-Net. The image surface shows lots of textural features, along with cracks; still, the trained network could detect the cracks in pixel-level. There is a high visual correspondence between the cracks in the original image and that in the detected ones. The time instants in Figure 10 are chosen such that they correspond to mentioned discrete jumps in the plots of the geometric measurements of Figure 8 and Figure 9. Towards the end of the experiment, the UAV is translated and rotated in such a way so as to capture the region of the concrete surface which remains occluded in iPhone video due to supporting columns of the Universal Testing Machine. Figure 11 shows the frame of the video, along with the tracked ROI, both for iPhone and UAV camera at 29.0 min after the start of the experiment. In this case, mobility of the UAV is harnessed to gain access to the concrete surface which remains hidden in ground-based iPhone video. The corresponding detected cracks are shown in the last row of Figure 10.

**Figure 12:**
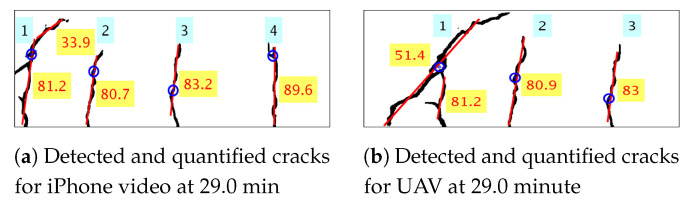
The red fitted line shows dominant crack orientation in degrees (red in yellow text box) of individual cracks which are numbered from left to right (black in cyan text box), for image frame at 29.0 min both for iPhone and UAV video.

Each frame of the video is processed to gather dense spatial geometric measurements of individual cracks. But, in Figure 8 and Figure 9, global measures to quantify the amount of damage due to crack in each frame of the video is plotted over time. Figure 12 shows individual detected cracks corresponding to the frames of the video both for iPhone, as well as UAV, as shown in Figure 11. The individual detected cracks are numbered from left to right in black fonts within cyan textboxes. Both the resultant images of Figure 12a,b processes part of the same region of the concrete surface with the cracks numbered as 2 and 3 are same in both the cases. The specific details of individual crack’s geometrical measurements are tabulated in Table 3. As previously discussed, the measurements of geometrical properties in physical units from video obtained from UAV will have errors. Still, the geometrical measurements corresponding to cracks numbered as 2 and 3 are almost similar for iPhone and UAV video.

#### 6.3.2. Crack Orientation

The qualitative orientation of crack is also of primary significance apart from its length, width and area for condition monitoring of concrete structures in order to assess the class of cracks. For beam elements, flexure cracks grow perpendicular upwards from beam edge in a qualitative sense, whereas shear cracks generally propagate at 45 degrees with respect to the beam edge. Merely fitting a line using least squares for individual crack is not sufficient as it will generate a single best fit line for cracks such as the ones shown in Figure 12b numbered 1. In reality, the referred crack is a flexure crack and shear crack joined together during their propagation. In addition, another example is crack numbered 1 shown in Figure 12a where a flexure crack while propagating turns diagonal midway through the depth of the beam and starts to propagate as a shear crack. Hence, successive RANSAC is used to obtain the dominant crack orientation of individual cracks as discussed in Section 5. The result of the successive RANSAC algorithm is shown in Figure 12. The best fitted straight line representing the dominant direction of crack propagation is shown in red, and the value of its orientation is shown in degrees (red text in a yellow text box).

**Figure 13:**
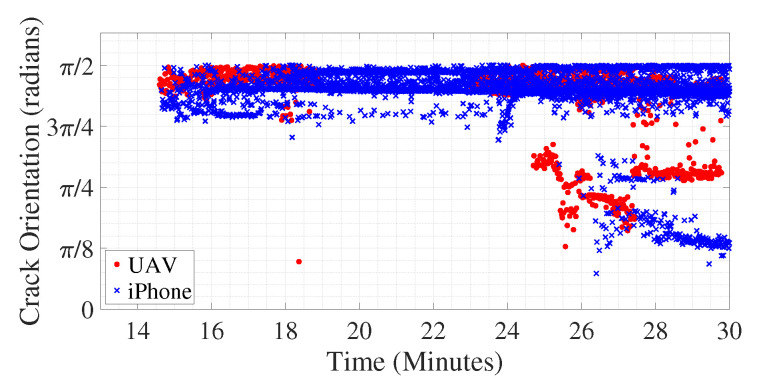
Dominant orientation of individual cracks in radians varying over time for video recorded using both iPhone and UAV.

Crack orientation is computed according to the above-mentioned method for all the frames of the video both for iPhone and UAV. The absolute values of the inverse tangent of the dominant slopes of all detected cracks in radians are shown in Figure 13 varying over time. As shown in Figure 8a, the first crack is detected around 14.5 min after the start of the experiment; hence, in Figure 13, the plot starts around that time. Initially, the orientation of the cracks is around π/2 radians, meaning the initial cracks propagates perpendicular to the beam’s lower edge, at its midspan, upwards, signifying flexural cracks as expected. However, when the UAV is moved so as to capture view of the beam away from its midspan, to the region of the concrete surface which is occluded in the video captured using iPhone, around 24.7 min after start of the experiment, diagonal cracks are detected in the video frames acquired using the UAV-based camera, as shown by presence of red diamonds around that time which are at an angle near π/4 radians. At that moment of time, the UAV-based camera could capture the left most diagonal crack, as shown in Figure 11b which remains occluded in Figure 11a. Over time, the diagonal crack propagates and gets visible in video captured using the iPhone camera; hence, there is a presence of blue crosses near crack orientation of π/4 radians towards the end of the experiment. This validates the efficacy of the above-mentioned method in computing the dominant direction of crack propagation which helps in identifying the nature of the crack. The beam failed around 30 min after the start of the experiment (see Figure 13), and the extraction of crack orientation and magnitude permits integrity assessment before failure occurs, which is the subject of future study.

## 7. Conclusions

The paper proposes an efficient method of processing video measurements from a camera mounted on UAV using deep neural network and Computer Vision algorithms to automatize the process of condition monitoring of concrete structures. Training a deep neural network image segmentation architecture called U-Net from a very small number of pixel-level annotated images is described. The trained model U-Net segments out pixels in the image belonging to cracks and converts the original RGB image of the concrete surface to binary image of cracks. The conversion of the original image to the binary image of the cracks is performed to compute the geometric properties of cracks, like length, width, area, and crack orientation, using morphological operations in which the steps are discussed in detail in the paper. In order to demonstrate the robustness and real-life applicability of the proposed framework, the laboratory experiment is carried out on an 8-foot-long beam which is gradually loaded until it fails in flexure. The images obtained from the video recordings of the experiment are not part of the dataset used for training a deep neural network; hence, it also demonstrates the robustness of the method, as well. In summary, the key contribution of the paper consists of the detection of cracks on the concrete surface, quantification of its geometric properties, like length, width, area, and dominant orientation, and inference of its nature. The paper experimentally validates the efficacy and robustness of the proposed framework as it tracks the propagation of cracks on its surface by successfully detecting multiple cracks on a full-scale concrete beam as it is loaded, measuring their geometric properties, as well as inferring the formation of initial formation of flexure crack and its evolution to shear cracks near its failure. The extracted pixel-level dense quantitative geometrical information, after detection of cracks propagating on the concrete surface, is valuable for recognizing the nature and extent of structural damage, thus enabling integrity assessment before failure occurs. The future extension of the proposed methodology includes the application of LiDAR installed on UAV for 3D mapping of the whole structure, as well as automatic segmentation of concrete surface from the acquired video. 

## Figures and Tables

**Table 1:** Hyper-parameters of U-Net: Batch Size and Number of epochs are selected according to their respective *Intersection-Over-Union (IoU) score*.

Batch Size	Number of Epochs	*IoU Score* of Crack Mask
5	4	0.5491
5	7	0.5769
5	10	0.5623
10	4	0.5181
10	7	0.5817
10	10	0.5430
10	20	0.6225
10	30	0.6366
10	40	0.6124

**Table 2:** Threshold level in Otsu’s method is selected according to *IoU score*, corresponding to the previously selected batch size and number of epochs.

Threshold Level	*IoU Score* of Crack Mask
0.75	0.6366
0.80	0.6817
0.85	0.6817
0.90	0.6798
0.95	0.6753

**Table 3:** Detailed geometric measurements of individual cracks as shown in Figure 11.

	Crack Number	Length (mm)	Area (mm${}^{2}$)	Maximum Width (mm)	Mean Width (mm)
**iPhone**	1	213.80	1243.10	7.35	3.92
	2	116.08	585.12	5.30	3.24
	3	125.63	562.99	4.16	2.97
	4	145.47	631.00	5.30	2.83
**UAV**	1	334.38	2145.61	16.41	4.62
	2	137.72	575.69	4.62	2.74
	3	115.94	491.57	3.62	2.69
